# Non-Alcoholic Fatty Liver Disease, Atherosclerosis, and Cardiovascular Disease in Asia

**DOI:** 10.31083/j.rcm2406173

**Published:** 2023-06-14

**Authors:** Yohwan Lim, Seogsong Jeong, Myunghee Hong, Hyun Wook Han

**Affiliations:** ^1^Department of Biomedical Informatics, School of Medicine, CHA University, 13488 Seongnam, Republic of Korea; ^2^Institute for Biomedical Informatics, School of Medicine, CHA University, 13488 Seongnam, Republic of Korea

**Keywords:** non-alcoholic fatty liver disease, cardiovascular disease, non-alcoholic steatohepatitis, metabolic dysfunction-associated fatty liver disease

## Abstract

The prevalence of non-alcoholic fatty liver disease (NAFLD) is estimated to increase to over half of the adult population by 2040 globally. Since the final diagnosis of NAFLD is made by a liver biopsy, several non-invasive approaches have been developed and validated to define NAFLD and evaluate NAFLD-associated diseases. Presently, NAFLD has been identified as an important and independent risk factor for developing several extrahepatic diseases, including atherosclerosis, cardiovascular disease (CVD), diabetes, and dementia. This review discusses current findings of up-to-date literature regarding the effects of NAFLD on the risk of atherosclerosis and CVD in Asia along with potential underlying biological mechanisms and therapeutic approaches to lower the NAFLD-related CVD risk. We further focus on the difference between NAFLD and metabolic dysfunction-associated fatty liver disease (MAFLD) on the risk of CVD and its implication by comparing the risk of NAFLD and MAFLD.

## 1. Introduction

Non-alcoholic fatty liver disease (NAFLD) is the most common etiology for the development of chronic liver diseases and end-stage liver diseases with increasing incidence worldwide [1]. The prevalence of NAFLD is estimated to increase to over half of the adult population by 2040 globally [2, 3]. NAFLD involves a spectrum that ranges from non-alcoholic fatty liver (NAFL) to non-alcoholic steatohepatitis (NASH), which is a condition of liver inflammation and hepatic damage that may contribute to the development of liver fibrosis, liver cirrhosis, hepatocellular carcinoma, and quality of life [4, 5, 6]. Treatment options for NAFLD are highly limited since no pharmacological therapy is approved for the treatment of NAFLD. Instead, lifestyle modifications, such as physical activity, weight control, and dietary change are suggested as the management of NAFLD [7, 8].

While the final diagnosis of NAFLD is made by a liver biopsy as a standard approach, several non-invasive diagnostic tests to define NAFLD have been developed and validated, such as imaging modalities, fatty liver index, hepatic steatosis index, and the Korean National Health and Nutrition Examination Survey NAFLD score [9, 10, 11, 12, 13]. The non-invasive approaches as a definition for NAFLD allowed a wider range of investigations on the association of NAFLD with both intrahepatic and extrahepatic diseases [13]. Presently, NAFLD has been identified as an important and independent risk factor for the development of several extrahepatic diseases, including atherosclerosis, cardiovascular disease (CVD), diabetes, and dementia [14, 15, 16, 17]. Herein, we reviewed up-to-date literature regarding the effects of NAFLD on the risk of atherosclerosis and CVD in Asia along with potential underlying biological mechanisms and therapeutic approaches to lower the NAFLD-related CVD risk.

## 2. Pathogenesis of NAFLD

**Fig. 1.**
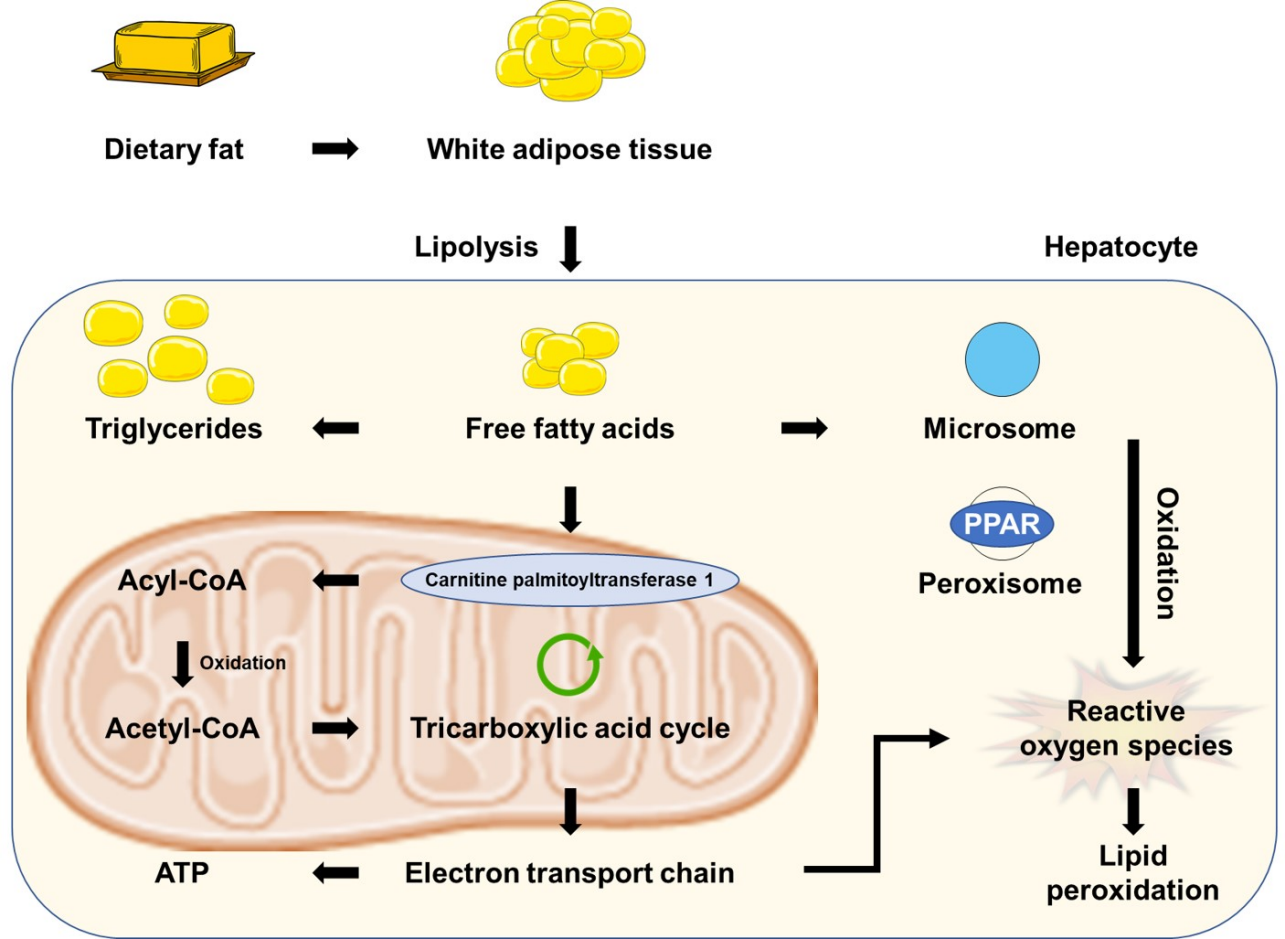
**The pathogenesis of NAFLD contributed by an ectopic accumulation of lipids and oxidative stress**. NAFLD, nonalcoholic fatty liver disease; ATP, adenosine triphosphate; PPAR, peroxisome proliferator-activated recepter.

The common clinical manifestations of patients with NAFLD include dysregulated lipid metabolism, such as the elevation of triglyceride and low-density lipoprotein cholesterol levels, which is considered a risk factor for incident CVD [18]. Although the comprehensive pathogenesis remains unclear, excess fatty acid-related insulin resistance and hepatic steatosis, subsequent lipid peroxidation, oxidative stress, and endoplasmic reticulum (ER) stress are being considered the pathogenesis of NAFLD [19]. The pathogenesis of NAFLD contributed by an ectopic accumulation of lipids and oxidative stress is shown in Fig. 1. The excessive triglyceride synthesis of hepatocytes from white adipose tissue, de novo lipogenesis, and dietary fat triggers hepatic steatosis in NAFLD [20, 21]. The excess free fatty acids are stored as triglycerides in the lipid droplets of white adipose tissue of the liver, leading to lipid ectopic deposits and NAFLD [22]. In addition, considering the anti-lipolysis effects, storage of triglycerides in adipose tissue, and promotion of esterification of insulin, insulin resistance is now being considered a key therapeutic factor against NAFLD [23]. Another pathogenesis of NAFLD is associated with ER stress, which activates the unfolded protein response to restore protein homeostasis [24]. ER homeostasis disruption has been detected in patients with NAFLD, suggesting a close association between ER stress and NAFLD [25].

NAFLD has also become the most common liver disease in children and adolescents, which may lead to serious implications, including both intra-hepatic and extra-hepatic morbidities, such as cardiovascular disease and cancer [26]. Fat accumulation, insulin resistance, gut-liver axis dysfunction, dietary factors, and genetic factors, including patatin like phospholipase domain containing 3, glucokinase regulator protein, apolipoprotein C-III, transmembrane 6 superfamily 2, and peroxisome proliferator-activated receptor gamma coactivator 1-alpha, are considered factors that are associated with the pathogenesis of NAFLD in children [27, 28].

## 3. Oxidative Stress in NAFLD

One of the important roles of reactive oxygen species (ROS) is cellular signaling, and oxidative stress occurs by an imbalanced production of ROS and the antioxidant scavenging capacity of the host [29]. In the liver, ROS signaling is considered a pro-determinant in the development of liver fibrosis [30]. Oxidative injury to nuclear DNA deteriorates mitochondrial function and disturbs nuclear-encoded mitochondrial gene transcription [31]. Nuclear factor erythroid 2-related factor 2, a modulator of antioxidant signaling that protects against oxidative stress, was found to be decreased in NAFLD [32]. In addition, a previous study indicated that the levels of urinary 8-iso-prostaglandin F2α and serum soluble nicotinamide adenine dinucleotide phosphate oxidase 2, which are markers of oxidative stress, were lower in patients with NAFLD [33]. These two markers were also revealed to be associated with the severity of steatosis detected by ultrasound. Lipid oxidation end products, such as 4-hydroxynoneal (4-HNE) and malondialdehyde, are also considered markers of oxidative stress. Podszun *et al*. [34] found that the level of 4-HNE adducts was significantly higher in NAFLD patients, and vitamin E treatment lowered the level of 4-HNE. In addition, they also suggested that quantification of 4-HNE by immunohistochemistry is assessable of hepatic lipid peroxidation. A recent study demonstrated that plasma pro-oxidative biomarkers are more related to the severity of NAFLD than nitrogen metabolism biomarkers measured in the liver tissue [35]. However, oxidative stress and nitrogen metabolism biomarkers are yet incomprehensively evaluated in terms of NAFLD-related atherosclerosis and cardiovascular disease. Considering the importance of the role of oxidative stress in NAFLD, atherosclerosis, and cardiovascular disease, biomarkers of oxidative stress may be useful in the prediction of NAFLD-related atherosclerosis and cardiovascular disease.

## 4. Association of NAFLD with Atherosclerosis

According to previous studies that reported the association of NAFLD with subclinical atherosclerosis as defined by increased carotid intima-media thickness, most studies indicated that NAFLD is associated with an increased risk of atherosclerosis, except for one study from Malaysia (Table 1, Ref. [36, 37, 38, 39, 40, 41, 42]) [36, 37, 38, 39, 40, 41, 42]. These results were in accordance with a previous study that defined premature coronary atherosclerosis as an outcome [14]. The most notable difference between these studies was that the prevalence of NAFLD was 57.4% in the Malaysian study as diagnosed using the Fibroscan, which is higher than the others [37].

**Table 1.** **Association of NAFLD with subclinical atherosclerosis as defined by increased CIMT in Asia**.

Study	Region	Year	Number of patients	Outcome	OR (95% CI)
Yi *et al*. [36]	China	2019	2745	CIMT >0.87	1.51 (1.04–2.18)
Tan *et al*. [37]	Malaysia	2018	251	CIMT >0.8	2.35 (0.77–7.21)
Zheng *et al*. [38]	China	2018	4112	CIMT >0.8	1.66 (1.39–1.99)
Huang *et al*. [39]	China	2012	8632	CIMT >0.8	1.32 (1.03–1.68)
Thakur *et al*. [40]	India	2012	80	CIMT >0.556	4.8 (1.8–12.8)
Kang *et al*. [41]	Korea	2012	633	CIMT >0.1	1.24 (1.02–1.47)
Kim *et al*. [42]	Korea	2009	1021	CIMT >0.8	1.63 (1.10–2.42)

OR calculated using logistic regression. Acronyms: CIMT, carotid intima-media thickness; OR, odds ratio; CI, confidence interval; NAFLD, nonalcoholic fatty liver disease.

Atherosclerosis has been considered to be involved by several systemic factors, including hypertension, diabetes, dyslipidemia, and lifestyle behaviors [43]. In addition, the reported underlying pathogenesis has been well-described in terms of inflammation, endothelial dysfunction, inflammation, and hemodynamic shear stress [44, 45, 46, 47, 48]. Patients with NAFLD often present with metabolic dysfunction, and thereby metabolic dysfunction-associated fatty liver disease (MAFLD) was proposed as a new definition of fatty liver disease which is defined according to the presence of hepatic steatosis, overweight or obesity, diabetes, and other metabolic abnormalities [49, 50]. Considering the common risk factors of NAFLD and atherosclerosis and the bidirectional contextual relationship between NAFLD and metabolic dysfunction, NAFLD-associated elevated risk of atherosclerosis may be associated with hepatic inflammation and systemic metabolic dysfunction [51].

## 5. Association of NAFLD and MAFLD with Cardiovascular Disease

Both CVD incidence and mortality are considered to be elevated by the presence of NAFLD or MAFLD [15, 52]. Hepatic inflammation, insulin resistance, and elevation of lipid levels are considered characteristics of NAFLD or MAFLD and were found to be associated with cardiovascular disease [15]. Recently, several researchers adopted a new clearer nomenclature with positive diagnostic criteria (MAFLD) in the evaluation of the risk of CVD and CVD mortality (Table 2, Ref. [15, 53, 54, 55, 56, 57, 58, 59, 60, 61, 62, 63]) [15, 53, 54, 55, 56, 57, 58, 59, 60, 61, 62, 63]. Unlike NAFLD, MAFLD includes patients with preexisting liver diseases, such as hepatitis B virus, and alcohol consumption, thus MAFLD criteria, which involve metabolic conditions, are considered more useful in the identification of patients who are at an increased CVD risk [50]. In addition, accumulating evidence suggested that the MAFLD definition is better than NAFLD in terms of patient management and alignment with other metabolic dysregulation-related diseases [54].

**Table 2.** **Events of CVD and CVD mortality according to the presence of NAFLD or MAFLD**.

Study	Outcome	NAFLD-only	MAFLD	No MAFLD
Jeong *et al*. [15]	CVD	340 (7.7)	5239 (12.5)	27,172 (9.3)
Guo *et al*. [53]	CVD	NR	308 (18.3)	882 (9.0)
Méndez-Sánchez *et al*. [54]	CVD	1 (3.4)	43 (6.2)	5 (1.8)
Lee *et al*. [55]	CVD	1248 (0.2)	101,188 (0.3)	81,235 (0.1)
Liu *et al*. [56]	CVD	NR	9181 (5.7)	6105 (2.3)
Liang *et al*. [57]	CVD	NR	162 (5.5)	134 (3.9)
Yoneda *et al*. [58]	CVD	NR	3002 (1.3)	10,455 (0.5)
Nguyen *et al*. [59]	CVD mortality	5 (2.0)	155 (5.7)	5 (2.0)
Kim *et al*. [60]	CVD mortality	11 (2.8)	228 (10.1)	348 (6.3)
Huang *et al*. [61]	CVD mortality	15 (2.8)	409 (10.5)	566 (6.6)
Semmler *et al*. [62]	CVD mortality	1 (1.4)	39 (1.8)	26 (1.0)
Liu *et al*. [63]	CVD mortality	NR	108 (3.3)	85 (2.6)

Data are n (%). Acronyms: CVD, cardiovascular disease; NAFLD, non-alcoholic fatty liver disease; MAFLD, metabolic dysfunction-associated fatty liver disease; NR, not reported.

The event rate of CVD and CVD mortality varies among different studies, potentially due to different durations of follow-up investigation and operational definitions of CVD. Jeong *et al*. [15] suggested that both hepatic steatosis and metabolic dysfunction were found to be unfavorable against incident CVD. The predictive effect for incident CVD was higher in MAFLD compared to sole NAFLD or metabolic dysfunction. A prospective cohort study from China also found that MAFLD elevated CVD risk after adjustments for cardiometabolic risk factors, including obesity, triglycerides, diabetes, dyslipidemia, and high-density lipoprotein cholesterol, suggesting patients with MAFLD to be monitored and to receive timely interventions to better manage CVD risks [43]. However, elevated CVD risk by MAFLD can be due to the inclusion criteria of alcohol consumption and concomitant liver diseases. MAFLD-only patients showed higher baseline metabolic dysregulations and “unhealthier” characteristics, and thus resulted in the highest CVD incidence rate compared to the combinations of NAFLD and/or MAFLD in several cohort studies [54, 55]. Since the new definition of MAFLD includes the coexistence of other liver diseases, it is evident that MAFLD-only patients showed the highest among other categories of MAFLD, which is supported by dual (or more) etiologies of fatty liver disease. The CVD risk by MAFLD is even amplified with high genetic risk scores of fatty liver disease-related genetic variants [56]. Therefore, further risk stratification models should be based on the combination of genetic and non-genetic variants, which represents the complex nature of the disease and its progression.

When comparing the risk of CVD, MAFLD had a higher risk of CVD than NAFLD. Prior studies proved that MAFLD and NAFLD were associated with higher risks of CVD [57, 58]. Furthermore, to estimate the difference between MAFLD and NAFLD definition, Lee *et al*. [55] compared the risk of CVD with the combination of NAFLD and/or MAFLD. The authors reported that the risk with NAFLD-MAFLD was the highest (hazard ratio (HR), 1.56; 95% confidence interval (CI), 1.54–1.58), followed by MAFLD-only (HR, 1.43; 95% CI, 1.41–1.45) and NAFLD-only being the lowest (HR, 1.09; 95% CI, 1.03–1.15). The cumulative CVD incidence was highest with MAFLD-only, followed by NAFLD-MAFLD and NAFLD-only. Another cohort study showed a higher risk of CVD with MAFLD-only (risk ratio (RR), 7.2; 95% CI, 2.4–21.5), but not with NAFLD-only (RR, 1.9; 95% CI, 0.25–14.8) [54]. The difference in the magnitude of risk may be explained by the small number of outcomes and discordance in measuring the presence of fatty liver within previous studies (e.g., ultrasound, fatty liver index).

The incidence rate of CVD mortality was also higher in MAFLD compared to NAFLD in most previous studies. A retrospective analysis showed significantly higher cumulative CVD-related mortality with MAFLD-only patients compared with NAFLD-only patients [59]. In addition, the risk of CVD mortality was higher with MAFLD-only patients compared with NAFLD-only and MAFLD-NAFLD patients, and rather showed “cardio-protective” effect with NAFLD-only patients [60, 61, 62, 63].

As expected, the definition of MAFLD captured more CVD-related outcomes than NAFLD. Since the new definition casts a “wider net” to include those with dual etiologies of fatty, MAFLD patients are more likely to have advanced fibrosis than those identified as NAFLD due to metabolic derangements [55, 64]. However, there are still controversies among the inclusion criteria of MAFLD. Even though there was a consensus reached to revise the terminologies to more accurately reflect the pathogenesis of NAFLD, the revised definition omits those with lean body mass index and without metabolic comorbidities, and therefore their potential risks are neglected. As there is rising concern regarding the potential CVD risks associated with lean NAFLD patients, the feasibility of the new definition needs to be interpreted with caution from a clinical perspective [50, 55, 65].

Cardiovascular risk factors, including obesity, metabolic syndrome, diabetes, hypertension, dyslipidemia, and chronic kidney disease, are also considered risk factors for NAFLD-related CVD [66]. The prevalence of NAFLD increased by elevation of blood pressure, and those with hypertension revealed a higher prevalence of advanced fibrosis of the liver [67]. Patients with diabetes also have a higher prevalence of NAFLD and NASH [68]. Currently, the coexistence of diabetes and NAFLD is considered to synergistically contribute to the disease progression [69]. However, whether glycemic control protects against the progression of NAFLD or ameliorates the disease remains unclear [70]. One of the features of NAFLD is dyslipidemia, including high-density lipoprotein cholesterol (HDL-C) dysfunction [71]. Previous studies have shown that a lower HDL-C level is associated with an increased risk of CVD, but clinical trials with the intent to lower the CVD risk by raising the HDL-C level have failed to prove beneficial effects [72]. Considering that cardiovascular risk factors are commonly present in NAFLD patients, management of cardiovascular risk factors may be crucial in lowering the risk of NAFLD-related CVD.

## 6. Potential Strategies to Lower the NAFLD-Related CVD Risk

Because there are no medications approved for the prevention or treatment of NAFLD, lowering the NAFLD-related CVD risks is challenging. Since NAFLD and CVD share several risk factors in common, early identification of NAFLD and prevention of its progression are crucial for lowering the risk of CVD. NAFLD barely manifests symptoms until it progresses, therefore routine annual check-ups of laboratory tests and abdomen ultrasonography will provide a higher early detection rate of NAFLD, which is recommended by many international guidelines [73, 74, 75]. In addition, those with a high risk of NAFLD including type 2 diabetes, metabolic syndrome, and dyslipidemia should be checked by transient elastography which has higher sensitivity and specificity against ultrasonography on finding and staging hepatic steatosis [73].

Management of metabolic risk factors is essential with NAFLD to reduce CVD risk. NAFLD, which is known as the hepatic manifestation of metabolic syndrome, is modulated by genetic and environmental factors including diet and lifestyle behaviors [76]. Therefore, managing dietary habits and environmental factors may stop the progression of NAFLD into activating inflammatory cascades by improving insulin resistance, reducing weight, and being active, supported by the “multiple hit” hypothesis [19]. The diet should not contain excessive fructose since it is a lipogenic and pro-inflammatory dietary factor that increases the development of NASH [77]. Moreover, some studies suggested Mediterranean diets, low in saturated fats and high in monounsaturated fatty acid, as a preferable dietary option for NAFLD patients as it has anti-inflammatory and antioxidant effects [78, 79]. For those who are overweight or obese, weight loss of 5% to 10% showed a histological improvement of NASH [80]. In addition, those who reduced their weight by more than 10% achieved a resolution of NAFLD, showing a dose-dependent association between weight loss and NAFLD improvement [81]. Bariatric surgery, which is recommended for severely obese patients (body mass index >40 kg/$m^{2}$) to reduce weight, showed long-term improvements in hepatic steatosis after the surgery [82, 83]. Recently, bariatric surgery showed a 49% risk-reduction of CVD compared with nonsurgical care [84]. Therefore, patients with severely obese NAFLD could benefit from bariatric surgery by significantly lowering CVD risk and improving NAFLD. Excise also showed an inverse relationship with NAFLD improvement in a dose-dependent manner [85]. Aerobic exercise decreases the hepatic fat and visceral fat mass, improving insulin sensitivity and reducing adiponectin [86]. Finally, smoking cessation needs to be achieved since smoking is a major risk factor for CVD. The association between smoking and NAFLD remains unclear, however, there were several studies suggesting a strong association between the two factors which needs further investigation on clarifying the underlying mechanism [87, 88].

Pharmacotherapy for NAFLD, which is focused on reducing hepatic steatosis and inflammation, is associated with reducing CVD risk in NAFLD patients. Targher *et al*. [69] proposed that the strong association between NAFLD and CVD is exacerbated by other underlying metabolic comorbidities such as type 2 diabetes and dyslipidemia, and therefore we could assume that controlling coexisting risk factors of CVD with other metabolic comorbidities may contribute to lowering the risk. An evidence-based practice guideline developed by the American Association for the study of Liver Diseases recommended the use of antioxidants (e.g., vitamin E), insulin-sensitizing agents (e.g., thioglitazone), and lipid-lowering drugs (e.g., statins) upon appropriate uses [75]. Moreover, sodium glucose cotransporter 2 (SGLT2) inhibitors and glucagon-like peptide 1 (GLP1) receptor agonists are reported to lower the serum liver enzymes, liver fibrosis index, and liver fat and should be considered for patients with type 2 diabetes and NAFLD [89, 90]. However, the evidence for using angiotensin-converting enzyme inhibitors and angiotensin receptor blockers in patients with hypertension and NAFLD is insufficient [91, 92].

## 7. Conclusions

NAFLD is identified as a significant risk factor for the development of atherosclerosis and CVD. Prior studies reported the association of NAFLD with increased carotid intima-media thickness, supporting the bidirectional relationship between NAFLD and dysregulated lipid metabolism. In addition, NAFLD increased the risk of incident CVD and CVD-related mortality. When evaluating the risk with the newer definition, MAFLD, the risk is elevated due to the wider definition of capturing higher baseline metabolic dysregulations and omitting healthier characteristics, when compared with NAFLD. Potential strategies to lower the NAFLD-related CVD risk may not be different from general therapeutic approaches to CVD.
